# Childhood adversities are different in Schizophrenic Spectrum Disorders, Bipolar Disorder and Major Depressive Disorder

**DOI:** 10.1186/s12888-018-1972-8

**Published:** 2018-12-19

**Authors:** Antonella Bruni, Elvira Anna Carbone, Valentina Pugliese, Matteo Aloi, Giuseppina Calabrò, Gregorio Cerminara, Cristina Segura-García, Pasquale De Fazio

**Affiliations:** 1 Psychiatry Unit, Department of Health Sciences, University “Magna Graecia” of Catanzaro, Viale Europa, 88100 Catanzaro, Italy; 2 Psychiatry Unit, Department of Medical and Surgical Sciences, University “Magna Graecia” of Catanzaro, Catanzaro, Italy

**Keywords:** Psychosis, Major depressive disorder, Bipolar disorder, Childhood adversities, Early life events

## Abstract

**Background:**

Research has shown that a history of childhood adversities is common in patients with psychiatric disorders but few studies have investigated links between specific types of adversity and specific psychiatric disorders.

**Methods:**

We investigated the frequency of early childhood adversities in a sample consisting of 91 patients with diagnosis of schizophrenic spectrum disorders (SSD), 74 patients with bipolar disorder (BD), 83 patients with major depressive disorder (MDD) and 85 healthy controls and sought to identify adverse early childhood life events that predict the development of major psychiatric disorders. The Childhood Experiences of Care and Abuse questionnaire was used to collect data on traumatic experiences occurring before the age of 17 years and comprehensive demographic data were also collected. The data were analyzed with chi-squared tests, t-tests, post-hoc and logistic regression.

**Results:**

Maternal absence/loss and economic difficulties in the early life were more prevalent in the BD group than other groups. Escape from home, cannabis abuse, psychological abuse, physical abuse and loneliness were more frequent in the SSD group than in other groups. Paternal absence, neglect of core needs, serious familial tension and absence of adult and peer confidants were all less common in the HC group than in the other groups. The regression model confirmed that different types of adversities play a crucial role in the development of the three investigated disorders.

**Conclusions:**

Our results support that SSD, BD and MDD are associated to different childhood adversities. This suggests that psychosocial interventions that reduce the incidence of these early life adversities might reduce the incidence of severe and disabling psychiatric disorders.

**Electronic supplementary material:**

The online version of this article (10.1186/s12888-018-1972-8) contains supplementary material, which is available to authorized users.

## Background

Studies have shown that distal risk factors, such as prenatal exposure to infection, obstetric complications, maternal micronutrient deficiencies [1–3] and stressful childhood life events are prevalent in patients with schizophrenic spectrum disorders (SSD) [4–6] and other mental illness such as mood disorders [7]. Amongst patients with psychosis the most frequent childhood adversities were emotional abuse, physical abuse and parental neglect [8, 9]. A comparison of subjects with no history of childhood abuse and subjects who had suffered abuse found that moderate and severe abuse were associated with, respectively, a seven-fold and forty-fold increase in risk of developing psychosis [10]. A recent meta-analysis concluded that rates of childhood adversity were similar in patients with schizophrenia, affective psychosis, depression and personality disorders [11]. A study comparing psychosis and unipolar depression found that specific types of adversity (i.e. emotional, psychological and physical abuse and parental separation) were related to these disorders; they were associated with a three-fold increase in risk of depression and a six-fold increase in risk of schizophrenia [12]. Both retrospective and prospective studies have demonstrated a strong association between childhood adversity and major depressive disorder (MDD) [13]. Furthermore, a meta-analysis concluded that patients with bipolar disorder (BD) were 2.6 times more likely to have experienced childhood adversity than healthy controls and that the most frequent form of abuse in this patient group was emotional abuse [14]. All these data suggest that there may be specific associations between forms of maltreatment and psychiatric disorders.

To the best of our knowledge, only one study compare Childhood Trauma Questionnaire (CTQ) scores between SSD, BD and MDD [15], whereas we have investigated a wider number of trauma in the present study. The first aim of this research was to assess the frequency of various forms of early childhood adversity in patients with SSD, BD and MDD. The second aim was to identify the associations between adverse early childhood life events and each of these three diagnoses.

## Methods

### Participants

The sample consisted of adults aged 18–65 years with a diagnosis of SSD, BD or MDD according to DSM-IV-TR criteria [16] who were enrolled at the Psychiatric Unit of the University Hospital Mater Domini, Catanzaro (Italy), between July 2015 and July 2017. Exclusion criteria were: 1) difficulty understanding the semi-structured questions; 2) history of a different psychiatric disorder from that currently being treated; 3) absence of a credible, documented medical history.

A control sample (HC) was also collected from the local community via Internet advertisements and from local university working staff and was chosen to reflect the group of patients based on age, social class and gender. Prior the assessment, they were all interviewed and asked about the lifetime presence of schizophrenia spectrum disorder or affective disorders and were excluded if so.

The final sample (*N* = 333) consisted of 91 patients with a diagnosis of SSD, 74 patients with BD, 83 patients with MDD and 85 HC individuals. Participants gave written informed consent to participation after receiving a full description of the aims and design of the study.

### Procedures

Participants first underwent a semi-structured interview designed to elicit socio-demographic information and information about psychiatric familiarity, prior psychiatric diagnoses, axis II comorbidity, previous suicide attempts, substance use and abuse began before the age of 17, significant life events (i.e. severe injury or disease to oneself or a close relative; death of partner; breakup of a relationship; a serious relational problem with a close friend/neighbor/relative; dismissal or difficulty in finding employment; significant financial or legal problems) in the 12 months prior to the onset of current psychiatric symptoms.

Diagnosis was made according the Structured Clinical Interview for DSM-IV-TR (SCID-I) [17]. The Italian version of “Childhood Experiences of Care and Abuse” was used to investigate traumatic episodes occurring before the age of 17 [18]. Cronbach’s alpha was 0.75 in this study.

The questionnaire is divided into three sections: a) composition of the birth family, parental attachments; b) any separation, abandonment or bereavement trauma; c) traumatic episodes that occurred before the age of 17 years (i.e. unscheduled change of school; expulsion from school; escape from home; periods in residential care; serious economic difficulties; neglect of major needs; parental conflict; psychological, physical and/or sexual abuse; lack of family and/or social support; loneliness). The subscale was dichotomized “yes/no” [see Additional file 1]. Assessments were conducted by trained psychiatrists or research assistants (masters- or doctoral-level clinicians). To assess inter-rater reliability, interviewer completed a mandatory training and only researchers that succeeded in passing the reliability checks were allowed to assess participants.

### Statistical analysis

A statistical power analysis was performed for sample size estimation. With an alpha = 0.05 and power = 0.85, the projected sample size needed with an effect size = 0.25 (GPower 3.1 or other software) is approximately *N* = 299 for this simplest between/within group comparison.

Data were analyzed using the Statistical Package for the Social Science, version 21.0 (SPSS Inc., Chicago Illinois) and are presented as frequencies and percentages (Fr, %).

Group differences were assessed with chi-square tests followed by Bonferroni post hoc as we used categorical variables. To identify independent predictors of each psychiatric diagnosis, statistically significant variables were entered into a forward stepwise multivariate logistic regression model with the following explanatory variables: adverse early childhood life events; age (continuous); gender (male = 0; female = 1); abuse of cannabis, cocaine, LSD, heroin or alcohol; adoption; parental death; parental estrangement; multiple changes of school; expulsion from school; escape from home; foster care; economic difficulties; neglect of core needs; family tension; psychological, physical or sexual abuse; absence of an adult confidant; absence of a peer confidant; loneliness (all variables: yes = 1; no = 0). Probabilities for stepwise entry and removal were 0.2 and 0.4. Type I error was set at *p* ≤ 0.05.

## Results

Table 1 describes the socio-demographic characteristics of the sample (Table 1). BD and MDD diagnoses were more frequent amongst women whereas SSD was the most frequent diagnosis amongst men. SSD patients were more likely to be single (SSD: 74.7%; BD: 28.4%; MDD 13.3%), unemployed (SSD: 51.6%; BD: 35.1%; MDD: 16.9%) and living with their parents (SSD: 62.6%; BD: 20.3%; MDD 13.3%). There were no group differences in educational level or educational attainment at 17 years.

**Table 1:** Sample description

	BD		DDM		HC		SSD		Statistics	P
	*N* = 74		*N* = 83		*N* = 85		*N* = 91			
Gender^a^										
Male	29	39.2	27	32.5	40	47.1	55	60.4	χ^2^ = 15.098	**.002**
Female	45	60.8	56	67.5	45	52.9	36	39.6		
Status civil^a^										
Single	21	28.4	11	13.3	22	25.9	68	74.7	χ^2^ = 116.344	**<.001**
Married	40	54.1	58	69.9	59	69.4	9	9.9		
Stable relationship	1	1.4	3	3.6	0	0.0	0	0.0		
Divorced	10	13.5	6	7.2	3	3.5	14	15.4		
Widower	2	2.7	5	6.0	1	1.2	0	0.0		
Education^a^										
Elementary school	9	12.2	17	20.5	6	7.1	9	9.9	χ^2^ = 17.960	.117
Middle school I	23	31.1	24	28.9	29	34.1	24	26.4		
High school II	27	36.5	31	37.3	32	37.6	44	48.4		
University degree	15	20.3	11	13.3	17	20.0	11	12.1		
Post graduate studies	0	0.0	0	0.0	1	1.2	3	3.3		
School performance at age 17^a^										
Low	38	51.4	45	54.2	40	47.1	44	48.4	χ^2^ = 13.266	.151
Average	10	13.5	15	18.1	8	9.4	17	18.7		
Good	20	27.0	18	21.7	22	25.9	25	27.5		
Excellent	6	8.1	5	6.0	15	17.6	5	5.5		
Employ^a^										
Unemployed	26	35.1	14	16.9	6	7.1	47	51.6	χ^2^ = 65.072	**<.001**
Unpaid job	22	29.7	27	32.5	24	28.2	18	19.8		
Student	2	2.7	6	7.2	3	3.5	6	6.6		
Part-time	5	6.8	9	10.8	14	16.5	11	12.1		
Full-time	19	25.7	27	32.5	38	44.7	9	9.9		
Family nucleus^a^										
Alone	10	13.5	4	4.8	7	8.2	10	11.0	χ^2^ = 111.47	**<.001**
Partner	42	56.8	61	73.5	57	67.1	9	9.9		
Parents	15	20.3	11	13.3	16	18.8	57	62.6		
Famil./Friends	7	9.5	6	7.2	5	5.9	7	7.7		
Structure	0	0.0	1	1.2	0	0.0	8	8.8		

^a^Frequency, %

*Abbreviations: BD* Bipolar Disorder, *MDD* Major Depressive Disorder, *HC* Healthy Control, *SSD* Schizophrenic Spectrum Disorder

For comparison, Chi-square test used for binary variables; the bold *P* values indicated the statistical significance

Table 2 shows the frequency of various early childhood events by group. Maternal separation and familial economic difficulties in early life were reported more frequently by BD patients than other groups whereas cannabis abuse, escape from home, psychological abuse, physical abuse and loneliness were more frequent in SSD patients than other groups. Paternal absence, neglect of core needs, serious familial tensions, lack of an adult confidant and lack of a peer confidant were all less frequent in the HC group than the clinical groups.

**Table 2:** Early Childhood events: comparison between groups and post-hoc

Early childhood events	HC *N* = 85		MDD *N* = 83		BD *N* = 74		SSD *N* = 91		χ^2^	p	Post-hoc
	Fr	%	Fr	%	Fr	%	Fr	%			
Cannabis Abuse	5	5.9	1	1.2	12	16.2	26	28.6	χ^2^ = 33.721	**<.001**	SSD > all; HC < all
Cocaine Abuse	0	0.0	0	0.0	4	5.4	6	6.6	χ^2^ = 10.695	.013	NS
LSD Abuse	0	0.0	0	0.0	0	0.0	4	4.4	χ^2^ = 10.767	.013	NS
Heroin Abuse	0	0.0	0	0.0	0	0.0	4	4.4	χ^2^ = 10.767	.013	NS
Alcohol Abuse	0	0.0	4	4.8	11	14.9	8	8.8	χ^2^ = 14.660	**.002**	NS
Absence of father	3	3.5	11	13.3	13	17.6	15	16.5	χ^2^ = 9.279	.026	HC < all
Absence of mother	2	2.4	6	7.2	13	17.6	8	8.8	χ^2^ = 11.853	**.008**	BD > all
Change of school	9	10.6	13	15.7	14	18.9	25	27.5	χ^2^ = 8.900	.031	NS
Home escape	0	0.0	4	4.8	11	14.9	20	22.0	χ^2^ = 27.056	**<.001**	SSD > all; HC < all
Serious economic difficulties	9	10.6	29	34.9	36	48.6	26	28.6	χ^2^ = 28.543	**<.001**	HC < all; BD > all
Neglect of major needs	3	3.5	18	21.7	20	27.0	21	23.1	χ^2^ = 17.935	**<.001**	HC < all
Serious family tensions	6	7.1	34	41.0	25	33.8	40	44.0	χ^2^ = 33.681	**<.001**	HC < all
Psychological Abuse	0	0.0	10	12.0	11	14.9	26	28.6	χ^2^ = 29.986	**<.001**	SSD > all; HC < all
Physical Abuse	1	1.2	12	14.5	18	24.3	38	41.8	χ^2^ = 46.849	**<.001**	SSD > all; HC < all
Absence of adult confident	18	21.2	38	45.8	36	48.6	47	51.6	χ^2^ = 20.465	**<.001**	HC < all
Absence of peer confident	10	9.7	25	21.1	25	33.8	43	47.3	χ^2^ = 26.271	**<.001**	HC < all
Experiences of loneliness	7	8.2	34	41.0	30	40.5	61	67.0	χ^2^ = 63.662	**<.001**	SSD > all; HC < all

*Abbreviations: HC* Healthy Control, *MDD* Major Depressive Disorder, *BD* Bipolar Disorder, *SSD* Schizophrenic Spectrum Disorder, *Fr* Frequency

For comparison, post hoc Bonferroni-corrected, Chi-square test used for binary variables; the bold *P* values indicated the statistical significance

Table 3 displays the results of the linear logistic regression models. Neglect of core needs, physical abuse, absence of a peer confidant and loneliness before 17 years old predicted SSD (−2Log-likelihood = 283.648; *p* < .001). Economic difficulties and the maternal separation or absence predicted BD (−2Log-likelihood = 330.395; *p* < .001). Not using cannabis and serious familial tensions were associated with the MDD diagnosis (−2Log-likelihood = 337.522; *p* < .001).

**Table 3:** Predictors of occurrence of Schizophrenic Spectrum Disorders, Major Depressive Disorder and Bipolar Disorder

Dependent variable	Independent variables	B	Standard Error	Wald	df	p	Exp(B)
SSD^a^	Neglect	−.924	.471	3.844	1	**.050**	.397
	Physical Abuse	.869	.403	4.637	1	**.031**	2.384
	Absence of peer confident	−.898	.335	7.194	1	**.007**	.407
	Loneliness	1.332	.331	16.215	1	**.000**	3.790
MDD^b^	Cannabis abuse	−2.486	1.044	5.666	1	**.017**	.083
	Serious family tensions	.828	.283	8.553	1	**.003**	2.290
BD^c^	Serious economic difficulties	.885	.286	9.613	1	**.002**	2.424
	Absence/separation of mother	.974	.416	5.486	1	**.019**	2.648

*Abbreviations: SSD* Schizophrenic Spectrum Disorder, *MDD* Major Depressive Disorder, *BD* Bipolar Disorder

^a^Model 1. Dependent variable: SSD; −2log Likelyhood = 283,648, *p* = <.001

^b^Model 2. Dependent variable: MMD; − 2log Likelyhood = 337,522; *p* = <.001

^c^Model 3. Dependent variable: BD; − 2log Likelyhood = 330,395; *p* = <.001

The bold *P* values indicated the statistical significance

## Discussion

Our results add to the evidence that stressful events in early life are related to the development of the major psychiatric disorders. To the best of our knowledge, this is the first study to have compared a wide number of childhood adversity in patients with SSD, BD and MDD.

We found that SSD was more frequent amongst men than women, whereas the most frequent diagnoses in women were BD and MDD. Gender differences in psychiatric disorders are a very controversial topic. Family adversity may be more stressful for girls vulnerable to psychosis than boys [19], but other data suggest than boys may be more vulnerable to the consequences of childhood adversity [6]. Another study of patients with psychosis and a history of childhood trauma found that men were more likely than women to report somatic or psychosomatic symptoms, including cardiovascular comorbidity, migraines and anhedonia, whilst women were more likely to report a lifetime history of elevated mood and being in a relationship [20]. Sex differences in these three disorders might be also explained by sex differences in emotional processing and coping strategies. It has been shown that males tend to react to trauma with hyperarousal, whereas females typically respond with dissociation [21]. Moreover, women tend to rely on adaptive coping styles when exposed to stress whereas men are more likely to use a fight-flight response [22, 23]. Our data are in line with a recent review [6] that showed worse overall functioning in SSD patients. Poor functioning has been also reported in the period before a first episode of psychosis and in individuals with subclinical psychotic symptoms and a history of childhood trauma [24]. Another study of first-episode psychosis patients [25] found that childhood adversity was associated with worse global functioning after the onset of psychosis but not in the premorbid period. Gil et al. [26] investigated whether specific types of childhood adversity differ in their effect on functional capacity in schizophrenia. They found that disability in schizophrenia is related to physical neglect, emotional abuse and neglect but not to other types of childhood trauma.

It is known that stressful situations represent one of the key triggers for psychosis and that stressful events are often the trigger for primary onset of psychosis and for subsequent relapses. Raune et al. concluded that stressful situations were more frequent in the 3 months prior to the onset of psychosis [27]. Similarly, we found that in our sample some stressful life events were associated to major psychiatric disease (i.e. SSD, BD and MDD) in later life. In particular, a history of physical abuse, at least 6 months of loneliness before 17 years, neglect of core needs and lack of a peer confidant were the variables associated with development of SSD. This result is in line with Xie et al. who found that SSD was related to higher emotional and physical scores on CTQ [15]. In a meta-analysis Varese et al. showed that childhood adversities (in particular sexual, physical and emotional abuse) were associated with a threefold increase in risk of psychosis (95% CI: 2.34–3.31) [28]. Unlike our study, this meta-analysis found no evidence for associations between specific types of adversity and psychosis. Morgan et al. found that people who reported both a history of childhood abuse and abuse of cannabis in the preceding year had a five times greater risk of experiencing psychosis than those who did not [29, 30]. A very recent meta-analysis of 44 studies on the relationship between childhood adversity and psychiatric disease concluded that non-specific childhood trauma, emotional abuse, physical neglect and high perceived stress are associated with SSD whilst sexual abuse, physical abuse and emotional neglect are not [6].

Contrariwise, our finding that premature loss of a parental figure is more common amongst BD patients than other groups is consistent with other studies which have reported an association between early parental loss and development of bipolar symptoms in adult life [31–33]. The mental health consequences of child maltreatment and child neglect have been carefully studied [34]. Other studies showed that emotional neglect was the only form of childhood adversity to differentiate BD patients from controls [35, 36]. In our sample, a referred emotional neglect is higher not only in patients with BD but also in the other groups (SSD and MDD).

Some studies have found associations between family tension, poverty and the development of depression in adulthood [37–40] but because they did not take into account confounders (environmental factors such as lack of educational and employment opportunities) they probably overestimated the strength of the associations [41]. We found that familial tension predicted MDD in adulthood whereas economic difficulties predicted BD.

Although this study has many strengths (i.e. sample size, comparisons between SSD, BD and MDD) it also has some limitations that must be addressed. The main limitation is the retrospective design. As our data on childhood trauma are retrospective self-reports the results may be influenced by recall bias. We tried to minimize recall bias by using a test/retest technique. Data were collected via a one-to-one patient interview, and then verified by the patient in the presence of a caregiver. Besides, a further limitation is that the study was restricted to the adverse events surveyed, rather than an open-ended survey. Finally, all forms of childhood adversities were represented as binary variables, we did not consider severity, duration or frequency; nor did we take into account the severity of current psychiatric symptoms. This last issue could be addressed in future studies. It would also be worth investigating other topics like: 1) the existence of specific protective factors in relation to major psychiatric disorders or 2) the possibility that childhood adversities could influence the age of onset of the psychiatric disorders rather than the diagnosis.

## Conclusions

The main purpose of this study was to compare history of early childhood adversity in patients with different psychopathological profiles. The results confirm that specific environmental factors seem to be associated to major psychiatric disorders. Some forms of childhood adversities, such as the neglect of major needs, physical abuse and loneliness, appear to play a crucial role in SSD, whereas maternal absence and familial economic difficulties resulted more strictly linked to the development of BD in later life. Family tension during childhood seems to be related to MDD. This suggests that psychosocial risk factors influence the development of psychiatric illness and suggests that psychosocial interventions targeting these factors could reduce the incidence of severe and disabling psychiatric disorders; if such a secondary prevention strategy for mental disorders were shown to be effective it would have important practical and social implications.

## Additional file

Additional file 1: Childhood Experiences of Care and Abuse, English version, EU-GEI. A set of questions investigating traumatic episodes occurring before the age of 17. (PDF 338 kb)

## Abbreviations

BD: Bipolar Disorder
CTQ: Childhood Trauma Questionnaire
DSM-IV-TR: Diagnostic and statistical manual, fourth edition, Text Revision
Fr: Frequencies
HC: Healthy Control
MDD: Major Depressive Disorder
SCID-I: Structured Clinical Interview
SD: Standard deviation
SPSS: Statistical Package for the Social Science
SSD: Schizophrenic spectrum disorder

## References

[1] Matheson SL, Shepherd AM, Laurens KR, Carr VJ (2011). A systematic meta-review grading the evidence for non-genetic risk factors and putative antecedents of schizophrenia. Schizophr Res.

[2] Brown AS (2011). The environment and susceptibility to schizophrenia. Prog Neurobiol.

[3] Brown AS, Derkits EJ (2010). Prenatal infection and schizophrenia: a review of epidemiologic and translational studies. Am J Psychiatry.

[4] Bendall S, Jackson HJ, Hulbert CA, McGorry PD (2008). Childhood trauma and psychotic disorders: a systematic, critical review of the evidence. Schizophr Bull.

[5] Walder DJ, Faraone SV, Glatt SJ, Tsuang MT, Seidman LJ (2014). Genetic liability, prenatal health, stress and family environment: risk factors in the Harvard adolescent family high risk for schizophrenia study. Schizophr Res.

[6] Fusar-Poli P, Tantardini M, De Simone S, Ramella-Cravaro V, Oliver D, Kingdon J (2017). Deconstructing vulnerability for psychosis: meta-analysis of environmental risk factors for psychosis in subjects at ultra high-risk. Eur Psychiatry.

[7] Uher R (2014). Gene-environment interactions in severe mental illness. Front Psychiatry.

[8] Cancel A, Comte M, Boutet C, Schneider FC, Rousseau P-F, Boukezzi S (2017). Childhood trauma and emotional processing circuits in schizophrenia: a functional connectivity study. Schizophr Res.

[9] Alameda L, Ferrari C, Baumann PS, Gholam-Rezaee M, Do KQ, Conus P (2015). Childhood sexual and physical abuse: age at exposure modulates impact on functional outcome in early psychosis patients. Psychol Med.

[10] Janssen I, Krabbendam L, Bak M, Hanssen M, Vollebergh W, de Graaf R (2004). Childhood abuse as a risk factor for psychotic experiences. Acta Psychiatr Scand.

[11] Matheson SL, Vijayan H, Dickson H, Shepherd AM, Carr VJ, Laurens KR (2013). Systematic meta-analysis of childhood social withdrawal in schizophrenia, and comparison with data from at-risk children aged 9-14 years. J Psychiatr Res.

[12] Rubino IA, Nanni RC, Pozzi DM, Siracusano A (2009). Early adverse experiences in schizophrenia and unipolar depression. J Nerv Ment Dis.

[13] Patten SB, Wilkes TCR, Williams JVA, Lavorato DH, El-Guebaly N, Schopflocher D (2015). Retrospective and prospectively assessed childhood adversity in association with major depression, alcohol consumption and painful conditions. Epidemiol Psychiatr Sci.

[14] Palmier-Claus JE, Berry K, Bucci S, Mansell W, Varese F (2016). Relationship between childhood adversity and bipolar affective disorder: systematic review and meta-analysis. Br J Psychiatry.

[15] Xie P, Wu K, Zheng Y, Guo Y, Yang Y, He J (2018). Prevalence of childhood trauma and correlations between childhood trauma, suicidal ideation, and social support in patients with depression, bipolar disorder, and schizophrenia in southern China. J Affect Disord.

[16] American Psychiatric Association. DSM-IV-TR: Diagnostic and Statistical Manual of Mental Disorders, 4th ed.Revised. 2000.

[17] First M, Spitzer R, Gibbon M, Williams J. Structured clinical interview for DSM-IV-TR Axis I disorders (SCID-I). in: New York, NY: New York State Psychiatric Institute Biometrics Research. 1995.

[18] van Os J, Rutten BP, Myin-Germeys I, Delespaul P, Viechtbauer W, European Network of National Networks studying Gene-Environment Interactions in Schizophrenia (EU-GEI) (2014). Identifying Gene-Environment Interactions in Schizophrenia: Contemporary Challenges for Integrated, Large-scale Investigations. Schizophr Bull.

[19] Gayer-Anderson C, Fisher HL, Fearon P, Hutchinson G, Morgan K, Dazzan P (2015). Gender differences in the association between childhood physical and sexual abuse, social support and psychosis. Soc Psychiatry Psychiatr Epidemiol.

[20] Sweeney S, Air T, Zannettino L, Galletly C. Gender Differences in the Physical and Psychological Manifestation of Childhood Trauma and/or Adversity in People with Psychosis. Front Psychol. 2015;6:1768. 10.3389/fpsyg.2015.01768 PMC4655246 26635676

[21] Read J, Perry BD, Moskowitz A, Connolly J (2001). The contribution of early traumatic events to schizophrenia in some patients: a traumagenic neurodevelopmental model. Psychiatry.

[22] Klein LC, Corwin EJ (2002). Seeing the unexpected: how sex differences in stress responses may provide a new perspective on the manifestation of psychiatric disorders. Curr Psychiatry Rep.

[23] Taylor SE, Klein LC, Lewis BP, Gruenewald TL, Gurung RA, Updegraff JA (2000). Biobehavioral responses to stress in females: tend-and-befriend, not fight-or-flight. Psychol Rev.

[24] Misiak B, Krefft M, Bielawski T, Moustafa AA, Sąsiadek MM, Frydecka D (2017). Toward a unified theory of childhood trauma and psychosis: a comprehensive review of epidemiological, clinical, neuropsychological and biological findings. Neurosci Biobehav Rev.

[25] Trauelsen AM, Bendall S, Jansen JE, Nielsen H-GL, Pedersen MB, Trier CH (2016). Childhood adversities: social support, premorbid functioning and social outcome in first-episode psychosis and a matched case-control group. Aust New Zeal J Psychiatry..

[26] Gil A, Gama CS, de Jesus DR, Lobato MI, Zimmer M, Belmonte-de-Abreu P (2009). The association of child abuse and neglect with adult disability in schizophrenia and the prominent role of physical neglect. Child Abuse Negl.

[27] Raune D, Kuipers E, Bebbington P. Stressful and intrusive life events preceding first episode psychosis. Epidemiol Psichiatr Soc. 18:221–8. 20034200

[28] Varese F, Smeets F, Drukker M, Lieverse R, Lataster T, Viechtbauer W (2012). Childhood adversities increase the risk of psychosis: a meta-analysis of patient-control, prospective-and cross-sectional cohort studies. Schizophr Bull.

[29] Morgan C, Reininghaus U, Reichenberg A, Frissa S, Hotopf M, Hatch SL (2014). Adversity, cannabis use and psychotic experiences: evidence of cumulative and synergistic effects. Br J Psychiatry.

[30] Morgan C, Gayer-Anderson C (2016). Childhood adversities and psychosis: evidence, challenges, implications. World Psychiatry.

[31] Etain B, Henry C, Bellivier F, Mathieu F, Leboyer M (2008). Beyond genetics: childhood affective trauma in bipolar disorder. Bipolar Disord.

[32] Laursen TM, Munk-Olsen T, Nordentoft M, Bo MP (2007). A comparison of selected risk factors for unipolar depressive disorder, bipolar affective disorder, schizoaffective disorder, and schizophrenia from a danish population-based cohort. J Clin Psychiatry.

[33] Mortensen PB, Pedersen CB, Melbye M, Mors O, Ewald H (2003). Individual and familial risk factors for bipolar affective disorders in Denmark. Arch Gen Psychiatry.

[34] Read J, van Os J, Morrison AP, Ross CA (2005). Childhood trauma, psychosis and schizophrenia: a literature review with theoretical and clinical implications. Acta Psychiatr Scand.

[35] Watson S, Porter RJ (2014). Childhood adversity in bipolar disorder. Aust New Zeal J Psychiatry.

[36] Etain B, Mathieu F, Henry C, Raust A, Roy I, Germain A (2010). Preferential association between childhood emotional abuse and bipolar disorder. J Trauma Stress.

[37] Nomura Y, Wickramaratne PJ, Warner V, Mufson L, Weissman MM (2002). Family discord, parental depression, and psychopathology in offspring: ten-year follow-up. J Am Acad Child Adolesc Psychiatry.

[38] Reinherz HZ, Giaconia RM, Hauf AM, Wasserman MS, Silverman AB (1999). Major depression in the transition to adulthood: risks and impairments. J Abnorm Psychol.

[39] Jaffee SR, Moffitt TE, Caspi A, Fombonne E, Poulton R, Martin J (2002). Differences in early childhood risk factors for juvenile-onset and adult-onset depression. Arch Gen Psychiatry.

[40] Spence SH, Najman JM, Bor W, O’Callaghan MJ, Williams GM (2002). Maternal anxiety and depression, poverty and marital relationship factors during early childhood as predictors of anxiety and depressive symptoms in adolescence. J Child Psychol Psychiatry.

[41] Weich S, Patterson J, Shaw R, Stewart-Brown S (2009). Family relationships in childhood and common psychiatric disorders in later life: systematic review of prospective studies. Br J Psychiatry.

