# Supplementary material for: Childhood adversities are different in Schizophrenic Spectrum Disorders, Bipolar Disorder and Major Depressive Disorder
Source: BMC Psychiatry. 2018 Dec 19;18:391. doi: 10.1186/s12888-018-1972-8 (PMC6300034; doi:10.1186/s12888-018-1972-8)
Supplement: Supplementary file 1 — Childhood Experiences of Care and Abuse, English version, EU-GEI. A set of questions investigating traumatic episodes occurring before the age of 17. (PDF 338 kb) [file 12888_2018_1972_MOESM1_ESM.pdf]

CHILDHOOD EXPERIENCES OF CARE AND ABUSE

I would now like to ask you some questions about your childhood and adolescence. We are interested in different experiences you may have had before you were 17 years of age. Some of the experiences I want to ask about may bring back upsetting or painful memories, so if at any time you do not wish to answer a question please say so. Of course, all information you provide will be treated in the strictest confidence.

1. Who were your main parent figures, before age 17? [If necessary, continue on a separate sheet]

**0 =**	**1 =**	**2 =**	**3 =**	**4 =**
**No mother, father figure**	**Natural mother, father**	**Step-mother, father**	**Grandmother, father**	**Other**

**1. Family arrangement**	**A. Mother figure**					**B. Father figure**					**C. Your age at start**
1. First	O0	O1	O2	O3	O4	O0	O1	O2	O3	O4	0
2. Second	O0	O1	O2	O3	O4	O0	O1	O2	O3	O4	
3. Third	O0	O1	O2	O3	O4	O0	O1	O2	O3	O4	
4. Fourth	O0	O1	O2	O3	O4	O0	O1	O2	O3	O4	
5. Fifth	O0	O1	O2	O3	O4	O0	O1	O2	O3	O4	
6. Sixth	O0	O1	O2	O3	O4	O0	O1	O2	O3	O4	

2. Did one or both of your parents die, before age 17?

2.a. Did your mother die? **O0 No** **O1 Yes**

2.b. Did your father die? **O0 No** **O1 Yes**

2.c. If yes, how old were you when your mother died? [ ][ ]

2.d. If yes, how old were you when your father died? [ ][ ]

3. Were you ever separated from a parent (longer than six months), before age 17?

3.a. Separated from mother? **O0 No** **O1 Yes**

3.b. Separated from father? **O0 No** **O1 Yes**

3.c. If yes, how old were you at your first separation from mother? [ ][ ]

3.d. If yes, how old were you at your first separation from father? [ ][ ]

3.e. How long were you separated, in months? [ ][ ]

3.f. What was the main reason for the separation?

O1 Parental Illness	O2 Divorce, Separation	O3 Work	O4 Never knew parent
O5 Own illness	O6 Boarding school	O7 Migration	O8 Other

3.g. Specify: ……………………………………………………………………………………………..

Before the age of 17 years …

	0-11 years			12-16 years		
**4.** Did you ever change schools? (other than change from primary to secondary)	O0 No	O1 Yes	O2 Refused to answer	O0 No	O1 Yes	O2 Refused to answer
**5.** Were you ever expelled from school?	O0 No	O1 Yes	O2 Refused to answer	O0 No	O1 Yes	O2 Refused to answer
**6.** Did you ever run away from home? (i.e., stayed away for more than two nights)	O0 No	O1 Yes	O2 Refused to answer	O0 No	O1 Yes	O2 Refused to answer
**7.** Were you ever taken into care? (i.e., children's home, fostered)	O0 No	O1 Yes	O2 Refused to answer	O0 No	O1 Yes	O2 Refused to answer
**8.** Were there ever times when your family was significantly short of money?	O0 No	O1 Yes	O2 Refused to answer	O0 No	O1 Yes	O2 Refused to answer
**9.** Were your basic needs ever neglected? (for food, clean clothing, etc.)	O0 No	O1 Yes	O2 Refused to answer	O0 No	O1 Yes	O2 Refused to answer
**10.** Were there ever frequent arguments or extreme tensions between your parents?	O0 No	O1 Yes	O2 Refused to answer	O0 No	O1 Yes	O2 Refused to answer
**11.** Were you ever tormented or treated cruelly by a parent or a member of household?	O0 No	O1 Yes	O2 Refused to answer	O0 No	O1 Yes	O2 Refused to answer
**12.** Were you ever hit or slapped on a number of occasions, sufficient to cause harm?	O0 No	O1 Yes	O2 Refused to answer	O0 No	O1 Yes	O2 Refused to answer
**13.** Ever had any unwanted sexual experiences?	O0 No	O1 Yes	O2 Refused to answer	O0 No	O1 Yes	O2 Refused to answer
**14.** Any adults could go to with problems or to discuss feelings?	O0 No	O1 Yes	O2 Refused to answer	O0 No	O1 Yes	O2 Refused to answer
**15.** Others own age could go to with problems or to discuss feelings?	O0 No	O1 Yes	O2 Refused to answer	O0 No	O1 Yes	O2 Refused to answer
**16.** Ever felt lonely for a significant period? (i.e., 6 months or more)?	O0 No	O1 Yes	O2 Refused to answer	O0 No	O1 Yes	O2 Refused to answer
